# Expression of the Hippo Pathway Core Components in Endometrial Cancer and Its Association with Clinicopathologic Features

**DOI:** 10.3390/diagnostics12122973

**Published:** 2022-11-28

**Authors:** Juseok Yang, Dae Hyun Song, Cho Hee Kim, Min Hye Kim, Hyen Chul Jo, Hyoeun Kim, Ji Eun Park, Jong Chul Baek

**Affiliations:** 1 Department of Obstetrics and Gynecology, Gyeongsang National University Changwon Hospital, 11, Samjeongja-ro, Seongsan-gu, Changwon-si 51472, Republic of Korea; 2 Department of Pathology, Gyeongsang National University School of Medicine and Gyeongsang National University Changwon Hospital, 11, Samjeongja-ro, Seongsan-gu, Changwon-si 51472, Republic of Korea; 3 Department of Obstetrics and Gynecology, Gyeongsang National University School of Medicine, Jinju 52727, Republic of Korea; 4 Department of Pathology, Gyeongsang National University Hospital, Jinju 52727, Republic of Korea; 5 Institute of Health Science, Gyeongsang National University, Jinju 52727, Republic of Korea

**Keywords:** endometrial cancer, Hippo pathway, YAP, *p*-YAP, LATS1/2, MST1/2, KIBRA, prognostic factors

## Abstract

Background: The Hippo signaling pathway has a key role in tumorigenesis. This study aimed to evaluate the relationship between the expression of core components of the Hippo signaling pathway and its association with clinicopathological features in endometrial cancer. Materials and Methods: We retrospectively collected endometrioid endometrial cancer specimens from 60 patients between January 2002 and December 2009 at Gyeongsang National University Hospital. Relevant clinicopathological data were obtained through electronic medical records of patients. The expression patterns of six core components (YAP, *p*-YAP, LATS1/2, MST1/2, KIBRA, and Merlin) were identified by immunohistochemistry on tissue microarray sections. Results: The positive expression ratio was 75.0% for YAP, 73.3% for *p*-YAP, 26.7% for MST1/2, 16.7% for KIBRA, 15.0% for Merlin, and 15.0% for LATS1/2. YAP expression was negatively correlated with MST 1/2 kinases (*p* = 0.045) and positively correlated with *p*-YAP (*p* = 0.012). Merlin, and MST 1/2 kinases (*p* = 0.043) showed a positive correlation. A subgroup of patients aged below 60 years (*p* = 0.004) and with myometrial invasion depth of less than 1/2 (*p* = 0.041) showed a positive association with YAP expression. *p*-YAP expression was negatively associated with a subset of patients with primary tumour size ≥4 cm (*p* = 0.03). Logistic regression analysis showed a significant association between age and YAP expression. The odds ratio of *p*-YAP expression was significantly lower in the group with tumour size ≥4 cm. Conclusion: Two prognostic factors, age and tumour size, were significantly associated with the expression of YAP and *p*-YAP in endometrial cancer. Further research should focus on their expression as a marker for prediction of clinicopathological implications in endometrial cancer.

## 1. Introduction

Endometrial cancer is the most common gynaecological malignancy [1]; its global incidence has gradually increased over the last 30 years, with high-income countries showing the greatest upsurge [2]. Although age-standardized global mortality has decreased over the same period, the mortality rate in some developed countries has increased [2]. Early diagnosis of many cases contributes to good prognosis, with a 5-year survival rate of 80–85% [3]. However, approximately 10% of patients with endometrial cancer who were early diagnosed and had a low-risk profile experience recurrence and deteriorating prognosis [4,5].

Adjuvant treatment approaches and follow-up plans are stratified based on the risk of recurrence that largely relies on the histologic findings of the tumour [6]. Recently, the ESMO-ESGO-ESTRO updated the consensus on defining prognostic risk groups, including molecular classification as a major variable [7]. To further improve stratifying strategy for the risk of recurrence in early-stage endometrial cancer, several biomarkers and signaling pathways have been proposed [8,9,10,11].

In 2003, the Hippo signaling pathway was first introduced as a tumour suppressor in the Drosophila model [12,13,14]. Later, convincing evidence supported the role of the Hippo-YAP pathway in angiogenesis [15,16], and more interestingly, mutations in the pathway-associated genes were demonstrated in some human cancer types [17,18,19]. The Hippo pathway consists of a cascade of serine/threonine kinases [17,20] and is well conserved in mammals [21]. It downregulates the activity of the transcriptional coactivators Yes-associated protein 1 (YAP) and transcriptional coactivator with PDZ-binding motif (TAZ) [21,22]. Despite numerous studies on the role of the Hippo pathway in angiogenesis, its carcinogenic role in human cancer cell models remains controversial. 

To date, the role of the Hippo-YAP pathway in gynaecological cancer is largely unknown. However, a few studies have elucidated its role in endometrial cancer [23]. In this study, we observed the expression levels of the Hippo pathway-associated genes in a tissue microarray (TMA) of a retrospective cohort of women with endometrial cancer. We aimed to examine the expression of the Hippo-YAP signaling pathway in human cancer and identify its potential as a prognostic molecular marker for predicting recurrence in endometrial cancer. 

## 2. Materials and Methods

### 2.1. Sample Collection

We retrospectively collected endometrioid endometrial cancer specimens from 60 patients who underwent hysterectomy, either by laparoscopy or laparotomy between January 2002 and December 2009 at Gyeongsang National University Hospital (GNUH), Jinju, Republic of Korea. Relevant clinicopathological data, including age at diagnosis, menopausal status, International Federation of Gynecology and Obstetrics (FIGO) staging, histologic grade, size of the tumour, lymphovascular space invasion (LVSI), and depth of myometrial invasion (MI), were obtained through electronic medical records of the patients. Two pathologists confirmed the diagnosis; cancer staging, and tumour histological type and grade were determined according to the Seventh Edition of the American Joint Committee on Cancer and the Fourth Edition of the World Health Organization classification, respectively. The Institutional Review Board of GNUH approved this study and waived the requirement o obtaining informed consent from patients (GNUH-2019-03-009). 

### 2.2. TMA and Immunohistochemical Stain

We selected typical sections of haematoxylin and eosin-stained slides that represent prominent intratumoral regions among the collected samples. From the invasive tumour front of each representative paraffin block, single core was obtained and transferred to a recipient TMA block.

Six types of immunostaining were performed on TMA using recommended doses of six different reagents according to the corresponding manufacturer’s instructions. Phospho-YAP (Ser127) (1:1000, monoclonal, D9W2I, #13008; Cell Signaling, Danvers, MA, USA), YAP (1:200, monoclonal, D8H1X, #14074; Cell Signaling, Danvers, MA, USA), KIBRA (1:200, polyclonal, ab216508; Abcam, Cambridge, MA, USA), LATS1/2 (1:200, polyclonal, #PA5-115498; Invitrogen, Carlsbad, CA, USA), Merlin (1:250, polyclonal, ab217016; Abcam, Cambridge, MA, USA), and MST1/2 (1:250, polyclonal, ab87322; Abcam, Cambridge, MA, USA) were used as primary antibodies. *p*-YAP, YAP, KIBRA, Merlin, and MST1/2 showed a diffuse staining pattern in the cytoplasm of cancer cells, whereas LATS1/2 showed a focal staining pattern in the cell nucleus. The staining level was read as 2-tiered. The stained tumour cells were graded as either positive or negative based on a higher intensity than that found in stromal cells and lymphocytes and an intensity equal to or lower than that of non-tumour cells, respectively. 

### 2.3. Statistical Analysis

The chi-square test and Fisher’s exact test were used for association analyses of categorical variables. Univariate and multivariate regression analyses were performed to determine the odds ratio of YAP and *p*-YAP expression based on the clinicopathological features of the cohort. To better understand the factors associated with Hippo molecules in endometrial cancer, backward stepwise elimination of the variables was conducted. The threshold value for variable selection was fixed at 0.2. Statistical significance was set at *p* < 0.05. The Spearman’ correlation coefficient test for measuring a linear correlation between two variables. The R square stat was performed in the logistic regression model and the stepwise regression model. The SPSS (ver. 24.0; IBM Corp., Armonk, NY, USA) and R software (ver. 4.21; R Project for Statistical Computing, Vienna, Austria) were used for statistical analysis.

## 3. Results

### 3.1. Patient Characteristics

The clinicopathological characteristics of the patients and the results of immunohistochemical staining are summarized in Table 1. The mean age of patients (*n* = 60) at diagnosis was 51.0 years, with more than 80% of patients (49/60, 81.7%) below 60 years of age. A total of 34 patients (56.7%) were menopausal at the time of diagnosis. Early endometrial cancer, including FIGO stage I (88.3%) and FIGO stage Ⅱ (3.3%), was 91.6%. Two-thirds of the tumours were of histologic grade 1 (40/60, 66.7%). Approximately 40% of cases were with tumour size larger than 4 cm. LVSI was positive in only 10% of the cases (6/60), and MI depth was less than 1/2 in 58.3% of the cohort (35/60).

### 3.2. Immunohistochemical Staining in Endometrial Cancer

**Figure 1:**
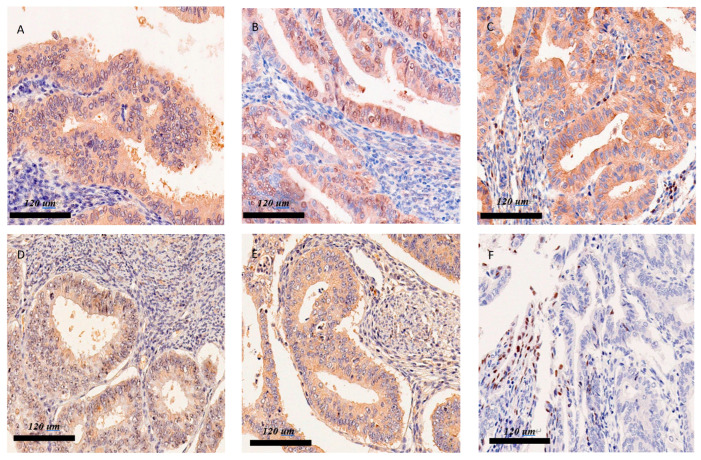
(**A**) Immunohistochemical staining: Cytoplasm of cancer cells shows diffuse expression of YAP (×400). (**B**) Cytoplasm of cancer cells shows diffuse expression of *p*-YAP (×400). (**C**) Diffuse expression of MST1/2 in the cytoplasm (×400). (**D**) Diffuse positive signal of KIBRA in the cytoplasm of cancer cells (×400). (**E**) Diffuse positive signal of Merlin in the cytoplasm of cancer cells (×400). (**F**) Diffuse positive signal of LATS1/2 in the cytoplasm of cancer cells (×400).

YAP and *p*-YAP were expressed in more than 70% cases of endometrioid endometrial cancer; excluding cases in which information was not available, 80% of the cases in this study were positive for YAP and *p*-YAP expression (80.3% and 80%, respectively; Figure 1A and Figure 1B, respectively). MST1/2 kinases were expressed in endometrial cancer cells in approximately a quarter of the cohort (26.7%). However, KIBRA antibody, Merlin protein, and LATS1 and LATS2 kinase were slightly expressed in tumour cells (16.7%, 15.0%, and 15.0%, respectively) (Figure 1C–F).

### 3.3. Association between YAP Expression and Core Proteins

The association analyses between Hippo pathway signaling regulators are shown in Table 2. There was a positive association between YAP and *p*-YAP as well as between Merlin and MST 1/2 kinases (*p* = 0.012 and *p* = 0.043, respectively). Although the expression levels of KIBRA, Merlin and LATS1/2 were not significantly associated with YAP expression in endometrial cancer, YAP and MST 1/2 kinases showed a negative correlation (*p* = 0.045). the expression levels of MST1/2, KIBRA, Merlin, LATS 1/2 kinase were not significantly associated with *p*-YAP expression in endometrial cancer. Regression analysis showed that the odds ratio of YAP and *p*-YAP expression levels were not altered by the expression of other molecules in the Hippo pathway. Only YAP and *p*-YAP were significantly associated with an OR of 8.13 (*p* = 0.009) in univariate regression analysis. 

### 3.4. Association between Clinicopathological Features and Expression of YAP, p-YAP, and LATS1/2

**Figure 2:**
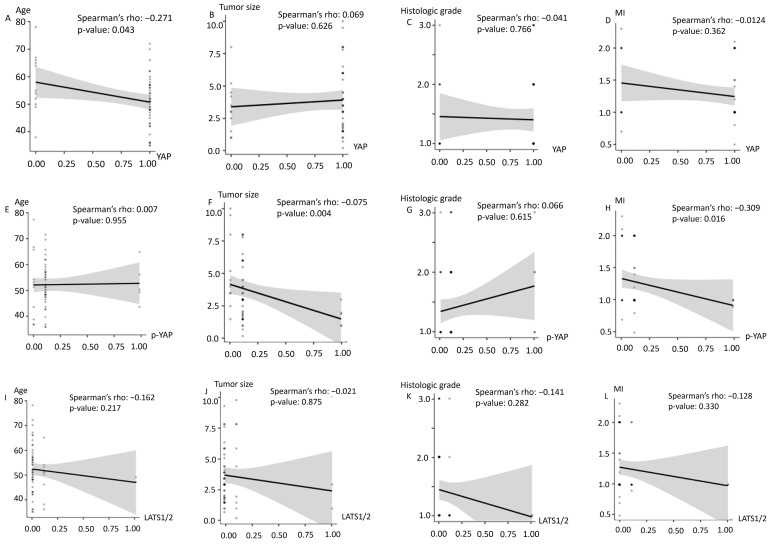
The Spearman correlation coefficient with the *p*-value. (**A,F,H**) showed an inverse correlation (Spearman correlation coefficient = −0.271, −0.375, −0.309 and *p*-value = 0.043, 0.004, 0.016, respectively). (**B**–**E**,**G**,**I**–**L**) showed no correlation between two variables.

The relationship between clinicopathological factors and the expression levels of YAP, *p*-YAP, and LATS1/2 is shown in Table 3 and its correlation coefficients with statistical significance of the coefficients are demonstrated in Figure 2. The overall result of each immunohistochemical staining differed due to the unavailability of information for a few samples. YAP was expressed more frequently in the subset of patients aged <60 years (*p* = 0.004) and with myometrial invasion depth of less than 1/2 (*p* = 0.041) showed a positive association with YAP expression. The status of menopause, FIGO stage of cancer, histologic grade, LVSI and the primary tumor size did not show any association with the expression of YAP protein. *p*-YAP showed a statistically significant association with a subset in which the primary tumour size was less than 4 cm (*p* = 0.03). No significant association was observed between LATS 1/2 expression level and clinicopathological features of the endometrial cancer patients. The relationship between YAP, *p*-YAP, LATS expression and clinicopathologic factors is demonstrated in Figure 2 with a trendline, including the correlation coefficients with statistical significance for all coefficients. Figure 2A,F,H showed monotonic correlation (Spearman correlation coefficient = −0.271, −0.375, −0.309 and *p*-value = 0.043, 0.004, 0.016, respectively).

### 3.5. Logistic Regression Analysis of YAP and P-YAP Expression

**Figure 3:**
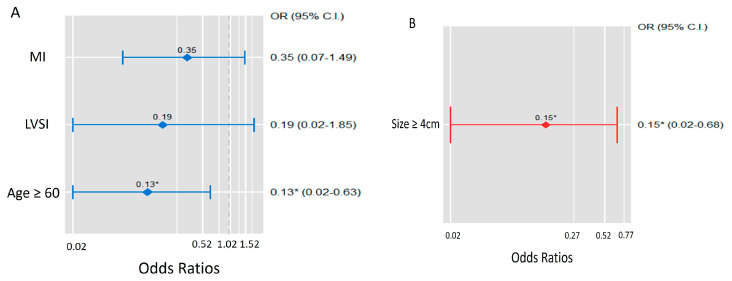
Visualization of odds ratios of YAP expression (**A**) and *p*-YAP expression (**B**) in endometrial cancer model. * Showed statistical significance.

Logistic regression analyses of YAP and *p*-YAP expression in endometrial cancer are shown in Table 4. There was a significant association between age and YAP expression in patients with endometrial cancer; patients aged >60 years had a lower chance of expressing YAP. Univariate, multivariate, and stepwise regression analyses showed statistical significance (OR 0.18 (*p* = 0.024), 0.14 (*p* = 0.033), and 0.13 (*p* = 0.013), respectively). Other variables did not alter the odds ratio of YAP expression in endometrial cancer. The tumour size was associated with the expression of *p*-YAP; the odds ratio of *p*-YAP expression was significantly lower at primary tumour size >4 cm. Moreover, univariate (OR 0.15, *p* = 0.026), multivariate (OR 0.14, *p* = 0.030), and stepwise regression (OR 0.15, *p* = 0.026) analyses showed statistical significance. The R square stat was performed in the logistic regression model and the stepwise regression model. R-squared value inserted in the Table 4. Figure 3 shows the odds ratio of the variables associated with the expression of YAP and *p*-YAP in stepwise backward elimination regression analysis.

## 4. Discussion

The Hippo signaling pathway has been demonstrated to be involved in the regulation of tissue growth and control of organ size and development [24]. Dysregulation of the Hippo pathway is well known to play a pivotal role in the development of several forms of cancer [25]. This is a large cascading network of serine/threonine kinases, including mammalian sterile 20-like kinases 1 and 2 (MST1 and MST2), large tumour-suppressor kinases 1 and 2 (LATS1 and LATS2), and neurofibromatosis type 2 (NF2)/Merlin. The mechanism by which the Hippo signaling pathway regulates tissue growth primarily depends on the phosphorylation of two transcriptional coactivator Yorkie (Yki) homologs, YAP and TAZ, which cannot directly bind to DNA. Phosphorylation of YAP/TAZ inhibits their translocation to the nucleus.

In humans, multiple intracytoplasmic signals, including KIBRA and Merlin, trigger the MST 1/2-LATS1/2 kinase cascade that eventually activates the phosphorylation of YAP/TAZ proteins. Phosphorylated YAP/TAZ recruits 14-3-3 proteins, which in turn generates cytoplasmic retention. In the nucleus, YAP/TAZ activates the transcription of genes that promote cell growth by binding to a transcription factor family of TEA domain family members (TEAD), and thereby controls cell proliferation and cell fate [26]. Target genes of the YAP/TAZ-TEAD complex include cysteine-rich angiogenic inducer 61 (CYR61) [27], connective tissue growth factor (CTGF) [28], and amphiregulin (AREG) [29,30]. Thus, it remains controversial whether the Hippo pathway is a tumour-suppressor or carcinogenic. 

Numerous studies have revealed the important role of the Hippo pathway in the tumorigenesis [17,18,19,24]. YAP and TAZ (downstream transcriptional co-activators) are oncogenes and can regulate tumorigenesis. The overexpression of YAP/TAZ is positively associated with uncontrolled cell growth and malignant transformation [17,18,24,31]. Abnormal activation of YAP/TAZ is detected in many solid tumours, including breast cancer, cholangiocarcinoma, colorectal cancer, gastric cancer, head and neck squamous cell carcinoma, and non-small cell lung cancer [23,24,25,26,31,32,33,34]. MST1/2 and LATS1/2 (upstream core kinases components) are important tumour-suppressors of the Hippo pathway. Decreased MST1/2 expression in colorectal cancer is associated with LN metastasis, vascular invasion, and poor prognosis. In gastric cancer, a higher clinical stage and LN metastasis is seen [33]. In breast cancer, colorectal cancer, gastric cancer, and lung cancer, LN metastasis is associated with decreased LATS1/2 expression [31,32,33,34]. In squamous cervical carcinoma and ovarian malignancy, decreased LATS1/2 expression is associated with higher clinical stage, disease recurrence, and poor prognosis [35,36]. 

Our study showed that YAP is extensively expressed in endometrial cancer. Immunohistochemical studies of the study cohort showed that other Hippo pathway-related molecules were not highly expressed. Interestingly, there was a significant association between the primary tumour size (with a cut-off of 4 cm) and the expression of *p*-YAP. While phosphorylation of YAP/TAZ (Hippo on) leads to proteasomal degradation and cytoplasmic retention, active YAP/TAZ (Hippo off) can translocate to the nucleus and stimulate cell proliferation by forming a complex with the TEAD transcription factor. Regression analyses showed a difference in the odds ratio of YAP and *p*-YAP expression in endometrial cancer. Surprisingly, YAP was only slightly expressed (0.13–0.18 times lower) in patients aged more than 60 years, suggesting a role of YAP and *p*-YAP in cell growth and survival. Additionally, when the primary tumour size was >4 cm, *p*-YAP was less frequently expressed in tumour cells. Notably, the correlation between the role of the Hippo pathway and clinicopathological features of endometrial cancer highlights its potential as a marker for predicting recurrence. The association between YAP and *p*-YAP expression, which showed higher odds ratios in univariate regression analysis, may be interpreted as consistent with the results of our cohort.

In this study, we investigated the association between Hippo pathway proteins. To our knowledge, the association between clinicopathologic factors and expression of the Hippo pathway upstream modulators was first reported in our study. There was a positive association between YAP and *p*-YAP as well as between Merlin and MST 1/2 kinases. Merlin triggers the phosphorylation cascade of MST and LATS, eventually leading to the phosphorylation of YAP/TAZ. However, the association between *p*-YAP and Merlin was not statistically significant in our study. The overall low expression level of Merlin, where only nine stained samples were positive (18.3%, excluding 11 cases with unavailability of information), might be considered for interpretation. Notably, LATS 1/2 did not show any association with other Hippo pathway molecules, and LATS 1/2 was expressed in the nucleus rather than the cytoplasm of cancer cell. 

The Cancer Genome Atlas (TCGA)’s study of Endometrial cancer patients showed frequently amplified in YAP/TAZ, WWTR1, and STK3 [37]. High expression levels of TAZ and low expression levels of LATS1/2 could be an important factor in the poor prognosis of cervical cancer. Pverexpression of YAP1 and under expression of LATS2 in ovary cancer also had poor prognosis. Dysregulation of the Hippo pathway could be an important factor in the poor prognosis of ovarian cancer and cervical cancer but not endometrial cancer [35,36,38]. 

Although Stage IA endometrial cancer showed an excellent 5-year overall survival rate of 90.3% [39,40], unexpected recurrence occurred in a substantial number of patients. Endeavors to recognize independent risk factors associated with disease recurrence in early-stage endometrial cancer are ongoing, including biochemical markers and genetic factors. The Hippo signaling pathway has potential as a prognostic marker for identifying the risk of recurrence in early-stage endometrial cancer, along with precise risk stratification.

Recently, Ma et al. demonstrated that LATS 1/2 stimulated tumour growth in ERα-positive breast cancer with ERα expression [41]. They showed that LATS 1/2 deficiency leads to a considerable decrease in the expression of estradiol induced transcribed ESR1 and Esα target genes. Ma et al. elucidated that the maintenance of ERα expression was supported by LATS 1/2, inconsistent with the previous report of Britschgi et al. [42] revealing that Hippo kinases LATS 1/2 cross-talked with ERα signaling and reduced ERα in a proteasomal degradation manner. 

The so-called Yin-Yang dynamics in the Hippo-YAP pathway [43] indicate that it behaves ambivalently as both tumorigenic and tumour suppressor, hindering our understanding and clinical application in predicting disease recurrence. In the present study, we showed the extensive expression of Hippo signaling pathway regulators in endometrial cancer and identified statistically significant associations between signaling proteins. Similar to conflicting roles of LATS 1/2 in ERα-positive breast cancer [44], LATS 1/2 kinases did not show consistent results with other regulators in our study. Indeed, we observed an extensive expression of LATS 1/2 expressed in the cell nucleus in endometrial cancer. Since the core pathway of the YAP/TAZ cascade, including the activation of upstream MST and LATS kinases, occurs in the cytoplasm [20], and translocation of YAP/TAZ to the nucleus is the final regulatory step, the finding of intra-nucleonic expression of LATS 1/2 necessitates further investigation. 

YAP shows a tissue-specific pattern of localization with nuclear or cytoplasmic expression. While increased cytoplasmic YAP staining is associated with LN metastasis and disease recurrence in cervical squamous cell carcinoma, high levels of nuclear YAP are associated with LN and distant metastasis in endometrial cancer [45,46]. Our study showed YAP was expressed within the cytoplasm of endometrial cancer cell. Since the expression pattern was observed in the primary tumour, not in the metastatic tissue, it is possible that YAP is only upregulated in the cytoplasm. Further work to discriminate localization with nuclear or cytoplasmic and its implication for regulation in tumorigenesis is needed.

This study had several limitations. First, the study had a relatively small sample size with some non-informative cases. Second, our cohort did not solely consist of early-stage endometrial cancer. In addition, the relationship between the Hippo pathway and disease recurrence is unclear. The study patients showed a good prognosis, and some patients received adjuvant treatment. All patients showed no recurrence; therefore, 5-year disease-free survival and overall survival rates could not be analyzed. 

## 5. Conclusions

Our study showed that there is a significant association between Hippo pathway core proteins in endometrial cancer and that the primary tumour size, with a cut-off of 4 cm, is associated with the expression of *p*-YAP. YAP was expressed more frequently in a subset of patients aged < 60 years. Considering the contradictions of LATS 1/2 kinases in human cancer, further research should be conducted on the expression of LATS 1/2 in endometrial cancer cell lines. 

## Figures and Tables

**Table 1:** Clinicopathological features in patients with endometrial cancer (*n* = 60).

Variable		Value
Age, years (mean [range])		51 [35–78]
<60 years, *n* (%)		49 (81.7)
≥60 years, *n* (%)		11 (18.3)
Menopause, *n* (%)	No	26 (43.3)
	Yes	34 (56.7)
FIGO stage, *n* (%)	1A	42 (70.0)
	1B	11 (18.3)
	2	2 (3.3)
	3A	2 (3.3)
	3B	1 (1.7)
	3C	2 (3.3)
Histologic grade, *n* (%)	1	40 (66.7)
	2	15 (25)
	3	5 (8.3)
* Tumour size, *n* (%)	<2 cm	16 (28.6)
	≥2 cm and <4 cm	18 (32.1)
	≥4 cm	22 (39.3)
LVSI, *n* (%)	Negative	54 (90.0)
	Positive	6 (10.0)
Myometrial invasion, *n* (%)	<1/2	35 (58.3)
	≥1/2	25 (41.7)
† YAP expression, *n* (%)	Negative	11 (18.3)
	Positive	45 (75.0)
	Non-informative	4 (3.7)
† *p*-YAP expression, *n* (%)	Negative	11 (18.3)
	Positive	44 (73.3)
	Non-informative	5 (8.3)
† MST1/2, *n* (%)	Negative	35 (58.3)
	Positive	16 (26.7)
	Non-informative	9 (15.0)
† KIBRA, *n* (%)	negative	45 (75.0%)
	positive	10 (16.7%)
	Non-informative	5 (8.3%)
† Merlin, *n* (%)	Negative	40 (66.7)
	Positive	9 (15.0)
	Non-informative	11 (18.3)
† LATS1/2, *n* (%)	Negative	49 (81.7)
	Positive	9 (15.0)
	Non-informative	2 (3.3)

FIGO: International Federation of Gynecology and Obstetrics; LVSI: Lymph vascular surface invasion; YAP: Yes-associated protein 1; *p*-Yap: Phosphorylated Yes-associated protein 1; MST: Mammalian STE20-like kinase; KIBRA: Kidney and brain protein; Merlin—also called Neurofibromin 2; LATS: Large tumour-suppressor kinase. * The primary tumour size was unmeasurable in four cases. † Non-informative samples exist because of tissue loss in tissue microarray block.

**Table 2:** Association between expression levels of YAP and *p*-YAP and other core proteins of Hippo pathway.

		YAP					*p*-YAP				
		Negative	Positive	*p* Value	Univariable OR (95% CI)	*p* Value	Negative	Positive	*p* Value	Univariable OR (95% CI)	*p* Value
* MST1/2 *n* (%)	Negative	8 (100.0)	27 (62.8)	0.045	-		8 (80.0)	27 (65.9)	0.474	2.07 (0.44–14.99)	0.394
	Positive	0 (0.0)	16 (37.2)				2 (20.0)	14 (34.1)			
KIBRA *n* (%)	Negative	8 (100.0)	35 (77.8)	0.327	-		10 (90.9)	33 (78.6)	0.667	2.73 (1.69–7.06)	0.368
	Positive	0 (0.0)	10 (22.2)				1 (9.1)	9 (21.4)			
* Merlin *n* (%)	Negative	7 (87.5)	33 (80.5)	>0.990	1.70 (24–34.07)	0.642	9 (90.0)	31 (79.5)	0.663	2.32 (0.35–46.03)	0.454
	Positive	1 (12.5)	8 (19.5)				1 (10.0)	8 (20.5)			
LATS1/2	Negative	8 (80.0)	39 (86.7)	0.627	-		8 (72.7)	39 (90.7)	0.140	-	
	Positive	2 (20.0)	6 (13.3)				3 (27.3)	4 (9.3)			
*p*-YAP *n* (%)	Negative	5 (55.6)	6 (13.3)	0.03	8.13 (1.72–42.38)	0.009	-				
	Positive	4 (44.4)	39 (86.7)								

YAP: Yes-associated protein 1; *p*-Yap: Phosphorylated Yes-associated protein 1; MST: Mammalian STE20-like kinase; KIBRA: Kidney and brain protein; Merlin—also called Neurofibromin 2; LATS: Large tumour-suppressor kinase. * MST and Merlin expression showed a significant correlation (*p* = 0.043).

**Table 3:** Association between clinicopathological features and expression of YAP, *p*-YAP, and LATS1/2.

		YAP Expression			*p*-YAP Expression			LATS1/2 Expression		
Clinicopathological Feature		Negative (*n* = 11)	Positive (*n* = 45)	*p* Value	Negative (*n* = 11)	Positive (*n* = 44)	*p* Value	Negative (*n* = 49)	Positive (*n* = 9)	*p* Value
Age	<60 years	5 (45.5%)	40 (88.9%)	0.004	8 (72.7%)	37 (84.1%)	0.400	39 (79.6%)	8 (88.9%)	>0.990
	≥60 years	6 (54.5%)	5 (11.1%)		3 (27.3%)	7 (15.9%)		10 (20.4%)	1 (11.1%)	
Menopause	No	3 (27.3%)	20 (44.4%)	0.496	4 (36.4%)	19 (43.2%)	0.745	20 (40.8%)	4 (44.4%)	>0.990
	Yes	8 (72.7%)	25 (55.6%)		7 (63.6%)	25 (56.8%)		29 (59.2%)	5 (55.6%)	
FIGO stage	1	10 (90.9%)	39 (86.7%)	>0.990	9 (81.8%)	39 (88.6%)	0.617	43 (87.8%)	8 (88.9%)	>0.990
	2.3	1 (9.1%)	6 (13.3%)		2 (18.2%)	5 (11.4%)		6 (12.2%)	1 (11.1%)	
Histologic grade	1	7 (63.6%)	41 (91.1%)	0.732	7 (63.6%)	31 (70.5%)	0.722	31 (63.3%)	7 (77.8%)	0.476
	2.3	4 (36.4%)	4 (8.9%)		4 (36.4%)	13 (29.5%)		18 (36.7%)	2 (22.2%)	
LVSI	Negative	9 (81.8%)	42 (93.3%)	0.251	9 (81.8%)	41 (93.2%)	0.259	45 (91.8%)	7 (77.8%)	0.231
	Positive	2 (18.2%)	3 (6.7%)		2 (18.2%)	3 (6.8%)		4 (8.2%)	2 (22.2%)	
MI	<1/2	3 (27.3%)	29 (64.4%)	0.041	4 (36.4%)	27 (61.4%)	0.180	27 (55.1%)	6 (66.7%)	0.718
	≥1/2	8 (72.7%)	16 (35.6%)		7 (63.6%)	17 (38.6%)		22 (44.9%)	3 (33.3%)	
* Tumour size (*n* = 52)	<4 cm	7 (63.6%)	23 (56.1%)	0.741	2 (18.2%)	22 (55.0%)	0.030	28 (62.2%)	4 (44.4%)	0.461
	≥4 cm	4 (36.4%)	18 (43.9%)		9 (73.8%)	18 (45.0%)		17 (37.8%)	5 (55.6%)	

Yap: Yes-associated protein 1; *p*-Yap: Phosphorylated Yes-associated protein 1; LATS: Large tumour suppressor kinase; LVSI: Lymph vascular surface invasion; MI: Myometrial invasion; FIGO: International Federation of Gynecology and Obstetrics. * The primary tumour size was unmeasurable in four cases.

**Table 4:** Logistic regression analysis of YAP and *p*-YAP expression in endometrial cancer.

Clinicopathologic Feature	YAP Expression (*n* = 56)						*p*-YAP Expression (*n* = 55)					
	Univariable OR (95% CI)	*p* Value	^(1)^ Multivariable OR (95% CI)	*p* Value	^(2)^ Stepwise † (95% CI)	*p* Value	Univariable OR (95% CI)	*p* Value	^(3)^ Multivariable OR (95% CI)	*p* Value	^(4)^ Stepwise † (95% CI)	*p* Value
Age ≥ 60	0.18 (0.04–0.81)	0.024	0.14 (0.02–0.80)	0.033	0.13 (0.02–0.63)	0.013	0.50 (0.11–2.72)	0.388	0.36 (0.04–2.71)	0.313	-	
Menopause	0.47 (0.09–1.86)	0.306	0.86 (0.11–6.46)	0.880	-		0.75 (0.18–2.87)	0.682	0.72 (0.13–3.96)	0.704	-	
Histologic grade 1	1.26 (0.29–4.93)	0.738	0.82 (0.15–4.02)	0.811	-		1.36 (0.31–5.36)	0.662	0.76 (0.13–3.68)	0.742	-	
LVSI	0.32 (0.05–2.70)	0.249	0.16 (0.01–1.69)	0.113	0.19 (0.02–1.85)	0.126	0.33 (0.05–2.77)	0.259	0.36 (0.04–3.74)	0.363	-	
MI	0.35 (0.08–1.32)	0.129	0.33 (0.06–1.48)	0.156	0.35 (0.07–1.49)	0.164	0.58 (0.15–2.19)	0.418	0.80 (0.18–3.58)	0.769	-	
* Tumour size ≥ 4 cm	1.15 (0.30–4.50)	0.838	1.46 (0.30–7.89)	0.640	-		0.15 (0.02–0.68)	0.026	0.14 (0.02–0.70)	0.030	0.15 (0.02–0.68)	0.026

Yap: Yes-associated protein 1; *p*-Yap: Phosphorylated Yes-associated protein 1; LVSI: Lymph vascular surface invasion; MI: Myometrial invasion. † Backward elimination using a threshold value of 0.2. * The primary tumour size was unmeasurable in four cases. R-squared value is 24.7% (1), 23.4% (2), 21.0% (3) and 12.6% (4), respectively.

## Data Availability

The datasets used and/or analyzed during the current study are available from the corresponding author upon reasonable request.
